# Exosomal LINC00853 promotes progression of gastric cancer via the MAP17/PDZK1/AKT signaling pathway

**DOI:** 10.1016/j.ncrna.2024.03.011

**Published:** 2024-03-30

**Authors:** Jung-ho Yoon, Hyo Joo Byun, Seo Yeon Kim, Da Hyun Jung, Sang Kil Lee

**Affiliations:** Department of Internal Medicine, Yonsei Institute of Gastroenterology, Yonsei University College of Medicine, Seoul, South Korea

**Keywords:** Exosomal lncRNA, linc00853, Early gastric cancer, Microarray, Carcinogenesis

## Abstract

Although rare, there is ongoing research into biomarkers that predict the onset and recurrence of gastric cancer, particularly focusing on substances found in exosomes. Long non-coding RNAs (lncRNAs) have garnered attention for their potential in diagnosing gastric cancer.

This study investigates the role of lncRNAs in gastric cancer, focusing on their presence in exosomes as potential biomarkers for the disease's onset and recurrence. We utilized the ArrayStar Human LncRNA array 2.0 to analyze lncRNA expression in tissues from early-stage gastric cancer patients. Our analysis highlighted LINC00853, which was significantly upregulated in cancer tissues and implicated in promoting epithelial-mesenchymal transition via the MAP17/PDZK1/AKT pathway. Functional studies on AGS and MKN74 gastric cancer cell lines demonstrated that LINC00853 facilitates cell proliferation, invasion, and migration. Additionally, RNA immunoprecipitation and electrophoretic mobility shift assays confirmed LINC00853 interaction with MAP17. Importantly, LINC00853 was also detected in exosomes from both patient samples and cell lines, and its downregulation led to decreased tumorigenicity in AGS cells. These findings suggest that both cellular and exosomal LINC00853 contribute to gastric cancer pathogenesis and may serve as valuable biomarkers for the disease.

## Abbreviations

EGC: early gastric cancer
lncRNAs: long non-coding RNA
EXO: exosome
qRT-PCR: quantitative real-time reverse transcription polymerase chain reaction
PBS: phosphate-buffered saline
SD: standard deviation
GTEx: genotype-tissue expression
TCGA: The Cancer Genome Atlas
EDS: EXO-depleted supernatant
CEA: carcinoembryonic antigen
CA 19-9: carbohydrate antigen 19-9
CA72-4: carbohydrate antigen

## 1 Introduction

Gastric cancer is one of the most lethal types of cancer of the gastrointestinal tract, despite a decrease in its incidence and mortality worldwide [1,2]. Since the national cancer screening program has become popular, patients with early gastric cancer (EGC) have a 5-year survival rate of over 90%. However, in most patients, gastric cancer would already have progressed to malignant proliferation and metastasis at the time of diagnosis [3,4]. Tumor markers, such as Carcinoembryonic antigen (CEA), Carbohydrate antigen 19-9 (CA 19-9), and Carbohydrate antigen 72-4(CA 72-4), are not preferred for gastric cancer screening because of their low sensitivity and false-positivity rates [5,6]. Therefore, there is a need for biomarkers that can specifically detect the early stages of the disease to help elucidate the mechanisms associated with metastasis and the occurrence of gastric cancer.

Long non-coding RNA (lncRNAs) are transcripts that contain more than 200 nucleotides and do not encode proteins. They have been shown to play important roles in various biological processes, including cancer development, by regulating gene expression through transcriptional regulation, post-transcriptional regulation, and chromatin modification [[7], [8], [9], [10], [11], [12]]. In addition, many studies have revealed that abnormal lncRNA expression contributes to tumor formation and metastasis [[13], [14], [15]]. However, studies on the discovery of lncRNAs for EGC detection are scarce.

Progression from adenoma to carcinoma in gastric tumorigenesis is expected to appear as a series of subsets, and multiple genetic alterations are expected to occur [16,17]. Epigenetic activation may result in early events that increase precancerous lesions, including metachronous recurrence, and may contribute to gastric tumorigenesis [[18], [19], [20], [21], [22]]. In earlier studies regarding gastric cancer, numerous lncRNAs have been proposed as biomarkers for high-risk gastric cancer through systematic evaluation and clinical analysis of metastasis [23]. While these endeavors have contributed to modifying our understanding of the complexes involved and identifying molecular mechanisms associated with gastric cancer progression [[24], [25], [26]], there remains a need for a focused molecular approach.

Extracellular vesicles are small lipid bilayer–enclosed vesicles that are released into body fluids, including blood, urine, and malignant ascites, with a size range of 20–200 nm [27,28]. These vesicles contain DNA, protein fragments, cytoplasmic microvesicles, and coding or non-coding RNA secreted from other cells. Recently, exosomes (EXO)—a subset of extracellular vesicles—have received considerable attention as components of new intercellular communication mechanisms among various cancer cells [29,30]. Cytoplasmic cell EXO can affect therapeutic approaches via the delivery of lncRNAs [31,32].

Microarrays have provided lncRNA and protein-coding mRNA expression data, allowing gene expression profiling in relevant co-expression and correlation studies [33]. Therefore, it is widely used for lncRNA detection and validation in various cancers, including breast [34], heart [35], bladder [36], liver [37], and prostate [38] cancers. However, the identification and cancer-related mechanisms of lncRNAs associated with the progression from EGC to progressive gastric cancer have not yet been elucidated.

In this study, we aimed to identify lncRNAs that are expressed at various stages of gastric cancer, including EGC, using a microarray. Additionally, we aimed to elucidate the associated mechanisms of action and confirm the potential application of such lncRNAs as EXO biomarkers. We also performed a study to elucidate the relationship between MAP17 and LncRNA. It has been established that MAP17 is overexpressed in various types of human cancers and enhances cancer cell proliferation, metabolism, invasion, and metastasis by augmenting ROS production and activating AKT signaling [39]. However, the impact and the specific mechanism of MAP17 in gastric cancer remain largely unknown. Therefore, our study provides novel insights into the role and regulation of MAP17 in gastric cancer.

## 2 Materials and methods

### 2.1 Gastric cancer tissue sampling

Gastric cancer tissues were collected from patients who had undergone endoscopic treatment for EGC and surgery for advanced gastric cancer. We used two types of patient tissues: (1) EGC samples obtained from 22 patients and (2) 100 pairs of tissues from patients with advanced gastric cancer for quantitative real-time reverse transcription polymerase chain reaction (qRT-PCR). (1) The microarray and EGC samples of 22 patients were approved for use by the Institutional Review Board (IRB) of Yonsei University College of Medicine. The experiments were conducted in accordance with the guidelines of the Helsinki Declaration and approved by the Public Institution Bioethics Committee designated by the Ministry of Health and Welfare and the IRB of Yonsei University College of Medicine (IRB number: 4-2013-0024; approval date: March 7, 2013). (2) The tissues used for qRT-PCR validation were collected before consent was obtained and were exempted from consent. A total of 100 samples were obtained from the Tissue Bank of the Research Institute of Gastroenterology, Yonsei University College of Medicine (Seoul, Korea). All patients had undergone gastric cancer surgery and had already completed treatment. No personal identification information was included in the clinical data or tissue samples; therefore, prior consent was not required. In patients with multiple cancers, tissues were collected from the largest lesions. In this study, we used tissues from six patients (a subset of the 22 patients with EGC; three with single gastric cancer and three with multiple gastric cancer) for microarray analysis. Additionally, 22 samples from patients with EGC who had undergone endoscopic biopsy and 100 samples from patients with advanced gastric cancer who had undergone surgery were used for qRT-PCR validation. The specimens collected by endoscopic biopsy were immediately stored in RNA later (Thermo Scientific, Rockford, IL, USA) and then frozen in liquid nitrogen and stored at −80 °C until use, and all collected surgical specimens were stored in liquid nitrogen.

### 2.2 Cell culture

Gastric cancer cell lines and normal cell lines used in this study were purchased from the Korean Cell Line Bank (KCLB, Seoul, Korea) and the American Type Culture Collection (ATCC, Rockville, MD, USA). For cell culture, RPMI-1640 medium or DMEM medium (Thermo Scientific) supplemented with 10% fetal bovine serum and 1% penicillin/streptomycin (Thermo Scientific) were used. All cells were cultured in appropriate culture conditions at 37 °C and 5% CO_2_ in a humidified atmosphere.

### 2.3 Small interfering RNA (siRNA) transfection

AGS and MKN74 cells (3 × 10^5^) were seeded in six-well plates and cultured in a 37 °C incubator for preparation for transfection. After 24 h, the cells were treated with siRNAs targeting each lncRNA (50 μM) or 50 μM control siRNA (siCT; Invitrogen, Carlsbad, CA, USA) using Lipofectamine 2000 reagent (Invitrogen) according to the manufacturer's protocol. The siRNA sequences used in the experiments are listed in Table 1.

**Table 1:** siRNA sequences for targeting lncRNAs.

Name	Direction	Sequence (5′ to 3′)
LINC00853_1	Forward	5′- GGAGGGCUCCCAGGACC -3′
	Reverse	5′- GGUCCUGGGAGCCCUCC -3′
LINC00853_2	Forward	5′- CCUGAUCUAAUGGAUCC -3′
	Reverse	5′- GGAUCCAUUAGAUCAGG -3′
MAP17	Forward	5′- GGAACAGAUGGAAGGUACU -3′
	Reverse	5′- AGUACCUUCCAUCUGUUCC -3′

### 2.4 Construction of the LINC00853 overexpression plasmid

LINC00853 was used to construct an overexpression vector using the pcDNA3.1(+) expression vector purchased from Addgene using the following cloning primer sequences: LINC00853_NheI_F (acccaagctggctagcGCCGCGCCTGAAGCTCAACT) and LINC00853_Xbal_R (aaacgggccctagaTGACACATGCAGAAATACTAT). The completed vector was transfected into AGS cells with Lipofectamine 2000 (Invitrogen) for 24 h with 1 μg of pcDNA3.1-LINC00853 to induce overexpression.

### 2.5 Mutagenesis assay

Mutation induction of the MAP17 binding site of LINC00853 was performed using a Q5 Site-Directed Mutagenesis kit (New England Biolabs Ltd., U.K.). The sequences used in this experiment were as follows: LINC00853-Mut_Antisense: CTGCTCTGAGCAAAATGGAAGACGATGGC, LINC00853-Mut_Sense: GCCATCGTCTTCCATTTTGCTCAGAGCAG. The LINC00853-Mut sequence information was confirmed by Sanger sequencing.

### 2.6 Total RNA extraction and qRT-PCR

Total RNA was isolated from tissues and cell lines using the TRIzol reagent (Invitrogen). RNA was quantified using a Nanodrop (ND-100; NanoDrop Technologies Inc., Wilmington, DE, USA) and confirmed by 1% agarose gel electrophoresis. Then, 2.0 μg of extracted RNA was used to synthesize cDNA using Superscript II (Invitrogen). LncRNA expression was measured by real-time PCR using iQ SYBR Green Supermix (Applied Biosystems Inc., Carlsbad, CA, USA). To normalize the Ct value of the sample, the expression of U6 or GAPDH was checked, and the final 2-ΔΔCt value was calculated. The primers used for qRT-PCR are listed in Table 2.

**Table 2:** Primer sequences for qRT-PCR.

Gene	Direction	Sequence (5′ to 3′)
LINC00853	Forward	5′-CAGAAAAGCTCCCGAAACTG-3′
	Reverse	5′-TTCCTTTGCCGGTAAAATTG-3′
LINC00634	Forward	5′-CTTGGAACTGGTGAGGGTGT-3′
	Reverse	5′-CATCTCATCTCCCCATGCTT-3′
LINC01535	Forward	5′-TGAATGCAGCTTTCTTGGTG-3′
	Reverse	5′-CAGGCTGATGGGGTATCTGT-3′
GAPLINC	Forward	5′-GTTTCCTGGAAGGGCATTTT-3′
	Reverse	5′-GTGCCTGAGTCCAGCTTCTC-3′
RP11-57A1.1	Forward	5′-GAGGCAACACTGCAGATGAA-3′
	Reverse	5′-AGAGGTTTCTGGCTGACTCG-3′
RP11-326C3.15	Forward	5′- GTGCTCCAGACCTTTTCCTG-3′
	Reverse	5′-TGCACCCATCAACACAGACT-3′
RP11-561O23.8	Forward	5′-GTGCTCCAGACCTTTTCCTG-3′
	Reverse	5′-TGCACCCATCAACACAGACT-3′
CYP4A22	Forward	5′-TTCAGCACGTCTCCTTGATG-3′
	Reverse	5′-ATGGCCTGGATGTAGGACTG-3′
MAP17	Forward	5′-TCAGGTCCAGTGAGCATGAG-3′
	Reverse	5′-ACTGGACATCCATCCCATGT-3′

### 2.7 Cell proliferation analysis

To investigate changes in the proliferation of gastric cancer cells, the cells were transfected with 50 μM siLINC00853, siMAP17, and siCT, and changes were measured from 0 to 72 h. This experiment was performed by CellTiter 96® AQueous One Solution Cell Proliferation Assay (Promega, Madison, WI, USA) in 96-well culture plates. Cells were allowed to react with the cell proliferation assay reagent (Promega) by incubation in the dark for 1 h and then measured using a spectrophotometer plate reader (Multiskan™ Microplate Photometer, Thermo Scientific) set at 490 nm. For the rescue experiment, 48 h after siRNA transfection, pcDNA-LINC00853 was transfected into gastric cancer cells, and cell proliferation was measured.

### 2.8 Cell cycle analysis

To confirm the cell cycle of gastric cancer cells, cells transfected with siRNA or siCT were washed with phosphate-buffered saline (PBS) and fixed with 75% ethanol at −20 °C overnight. The cells were resuspended in PBS to collect them and treated with RNase for 30 min at 25 °C. Cell nuclei were stained with 50 mg/mL propidium iodide (Sigma-Aldrich) for 10 min in the dark and analyzed using flow cytometry (BD Biosciences).

### 2.9 Scratch wound healing assay and invasion assay

To study the effect of regulating the expression of siLINC00853s and siMAP17 on wound healing, cells transfected with siRNA or siCT were re-inoculated into six-well plates. When the cells reached approximately 60% confluence, the bottom of the well was scraped evenly using a P20 tip. The width of the cells scratched by the tip was measured under a microscope at 0, 24, and 48 h. For invasion assays, the same cell lines and conditions described above were used and reproduced in BD BioCoat Transwells (BD Biosciences). After 24 and 48 h, non-invasive cells in the insertion chamber were carefully removed, and the upper layer of the Transwell was gently wiped with a cotton swab. The membrane at the bottom of the upper chamber was fixed with 5% acetaldehyde buffer. The cells were stained with a crystal violet solution and observed under a microscope. Subsequently, the number of invading cells was determined.

### 2.10 Soft agar colony formation assay

To analyze colony formation, we performed the experiment according to the protocol provided by CytoSelect™ 96-well cell transformation assay (CELL BIOLABS, INC, San Diego, CA, USA). The bottom and top agarose plates were coated onto 96-well culture plates. We filled each well with 1.5 mL of 2 × DMEM containing 1% agarose along with the base layer. After coagulating for 1 h, we dissolved the cells transfected with siRNA or siCT in 2 × DMEM containing 0.7% agarose and incubated them for 2–3 weeks in a 37 °C incubator. Colonies were observed under a microscope.

### 2.11 Western blot

Cells transfected with siRNA or siCT were dissolved in 1 × RIPA buffer (Cell Signaling Technology, Danvers, MA, USA) with protease inhibitors to inhibit protein degradation. The proteins were separated by centrifugation and loaded according to their size for experimental purposes using 8–15% sodium dodecyl sulfate polyacrylamide gel electrophoresis. For antibody analysis, the gel was transferred onto a polyvinylidene difluoride membrane (GE Healthcare, Piscataway, NJ, USA). We blocked the transferred membrane with 5% bovine serum albumin (BD biosciences) and 0.1% Tween 20 in trisphosphate buffer for 1 h at room temperature and then incubated it overnight with primary antibody according to the antibody conditions at 4 °C. After washing, we reacted the membrane with the secondary antibody according to each antibody condition and obtained the results using the Image Quant LAS 4000 biomolecule imager with ECL solution (GenDEPOT, Barker, TX, USA).

The following primary antibodies were used for Western blot analysis: E-cadherin (1:1000, BD biosciences); N-cadherin (1:1000, BD Biosciences); Vimentin (1:200, Santa Cruz Biotechnology, Texas, DA, USA, sc-373717); Snail (1:1000, Cell Signaling Technology, Danvers, MA USA, #3879S); PARP (1:1000, Cell Signaling Technology, #9542); Bcl-xl (1:1000, Cell Signaling Technology, #2764); Caspase-9 (1:1000, Cell Signaling Technology, 9504); MAP17 (1:1000, Abcam, ab156014); Phospho-Akt (Ser473) antibody (1:1000, Cell Signaling Technology, #12694); anti-PDZK1 (1:1000, Abcam, ab121248); anti-TSG101 (1:2000, Abcam, ab30871); anti-CD63 antibody (1:2000, Abcam, ab231975); LaminB (1:4000, Santa Cruz Biotechnology, sc-374015); and β-actin (1:5000, Bioworld Technology, Louis Park, MN, USA, AP0060). The signal was developed in ECL solution (GenDEPOT, Barker, TX, USA) and exposed to an ImageQuant LAS 4000 biomolecular imager for 2 min.

### 2.12 RNA immunoprecipitation (RIP)

The cells were resuspended in RIP buffer (Abcam) containing a mixture of RNase (GenDEPOT) and protease inhibitors (GenDEPOT). An ultrasonic processor was used for chromatin cutting. We performed 20 cycles (170–190 W) of shearing and repeated the cooling in a cooling bath to minimize heat loss. After centrifugation, we applied the antibody to the supernatant and cultured it overnight at 4 °C. The next day, we added 20 μL of magnetic beads (Millipore) and reacted the solution for 1 h at room temperature on a rotator. The cells were washed twice with RIP buffer, and RNA was extracted.

### 2.13 Electrophoretic mobility shift assay (EMSA)

MAP17 and LINC00853 binding activity were detected using an EMSA kit (Promega Corp., Madison, WI, USA). AGS cells were obtained using a Nuclear Extraction kit (Affymetrix, CA, USA). The experiments were performed according to the manufacturer's protocol. Briefly, nuclear proteins were hybridized with a double-stranded, biotin-labeled oligonucleotide probe containing a common binding site for MAP17 (sense strand, 5′-CGGAAACAAGGCAGATGGAGTCC-3′). The protein–DNA complex was separated on a 6% non-denaturing PAGE gel and transferred to a Pall Biodyne B nylon membrane (Pall Life Sciences, Pensacola, FL, USA). Signal detection was performed using streptavidin–horseradish peroxidase and a chemiluminescent substrate.

### 2.14 Exosome extraction, isolation, and treatment

ExoQuick® Exosome Isolation and RNA Purification Kit (System Biosciences, LLC, Palo Alto, CA, USA) was used, and we performed the experiment according to the manufacturer's protocol. This kit was used to extract EXO from gastric cancer cell culture media and patient plasma samples. We finally extracted 10 μL of pure RNA.

### 2.15 Transmission electron microscopy (TEM)

The exosome pellet was resuspended in 0.1 M sodium cacodylate buffer (pH 7.0) containing 5% glutaraldehyde and fixed for 1 h at 4 °C. A drop of the exosome suspension was placed on a Formvar-carbon coated EM grid and stained with 2% phosphotungstic acid solution (pH 7.0) for 30 s. The samples were observed with a transmission electron microscope (TEM: JEM-1011, JEOL, Tokyo, Japan, MegaviewⅢ).

### 2.16 Microarray analysis

For microarray analysis, we extracted RNA from tissue samples of 12 patients using TRIzol (Invitrogen). LncRNA expression analysis was performed using ArrayStar Human LncRNA array 2.0 (ArrayStar, Rockville, MD, USA). Candidates were selected for further analysis if the fold-change was > 2.0 and the P-value was < 0.05.

### 2.17 Statistical analysis

All data used in the experiment are shown as the mean ± standard deviation (SD) for all analyses of continuous variables. All statistical processes were performed using SPSS software (version 18.0, SPSS Inc., Chicago, IL, USA), and the tests included a *t*-test, χ2 test, Fisher's exact test, and a one-way analysis of variance test.

## 3 Results

### 3.1 Detection of five lncRNAs potentially involved in EGC onset and recurrence

**Fig. 1:**
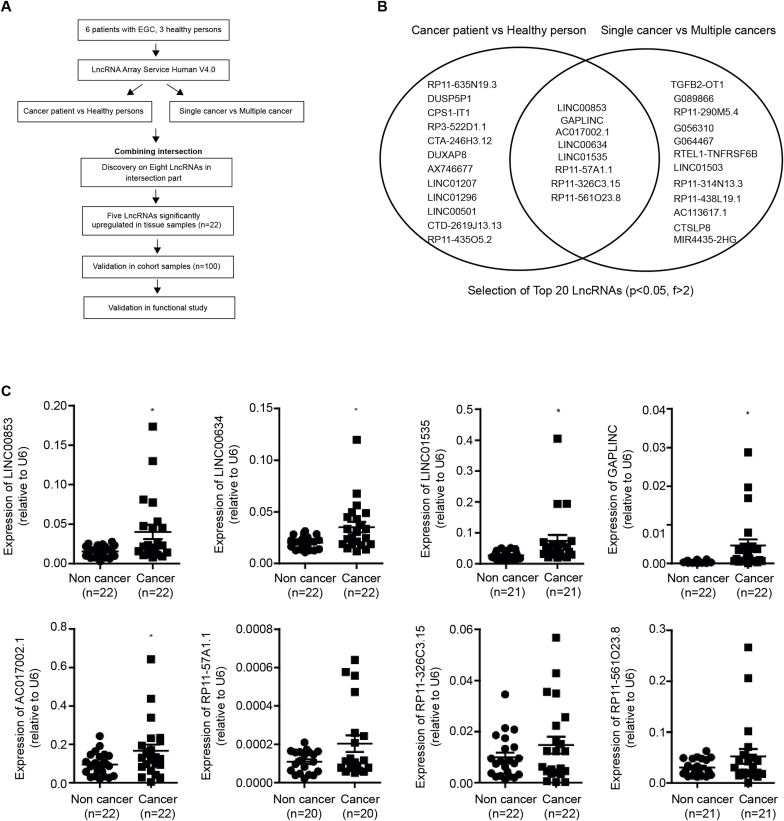
**Detection of lncRNAs in gastric cancer tissues.** (A) Schematic representation of lncRNAs derived from microarray workflow (fold change > 2, P ≤ 0.05). (B) Venn diagram of differentially expressed lncRNAs from microarray analysis. On the left, the comparison between patients with gastric cancer and healthy individuals is shown, and on the right, the comparison between single cancer and multiple cancers is shown (fold change > 2, P ≤ 0.05). (C) qRT-PCR validation of eight differentially expressed lncRNAs in 22 patients with EGC. LINC00853 expression was higher than LINC00634, LINC01535, GAPLINC, AC017002.1, RP11-57A1.1, RP11-326C3.15, and RP11-561O23 in gastric cancer tissues compared to that in adjacent normal tissues (n = 22).

We performed an lncRNA microarray analysis on six patients with EGC and three healthy individuals (Fig. 1a). Among the patients, three had a single EGC and three had multiple EGC—at least two simultaneous gastric cancers at the time of diagnosis. We compared the expression of lncRNAs between EGC tissues and normal tissues (from healthy individuals) to identify lncRNAs related to EGC development, and between non-cancerous adjacent tissues of patients with single and multiple EGC to identify lncRNAs related to multiple recurrences. To select lncRNAs involved in both EGC onset and recurrence, we applied the following criteria: (1) lncRNAs that were upregulated in cancer tissues compared to normal tissues, with a fold change > 2 and P-value ≤ 0.05; (2) lncRNAs that were upregulated in non-cancer adjacent tissues of patients with multiple EGC compared to those with single EGC, with a fold change > 2 and P-value ≤ 0.05; (3) the top 20 lncRNAs that met both conditions (1) and (2); (4) overlapping top 20 lncRNAs in each comparison (Fig. 1b). Eight candidate lncRNAs were identified, and their expression levels were validated by qRT-PCR using cancer and non-cancer adjacent tissues from 22 patients with EGC. The expression levels of LINC00853, LINC00634, LINC01535, GAPLINC, and AC017002.1 were significantly higher in gastric cancer tissues than in non-cancerous adjacent tissues (P ≤ 0.05), confirming that these five lncRNAs were differentially expressed in the EGC samples. RP11-57A1.1, RP11-326C3.15, and RP11-561O23.8 did not show any significant differences (Fig. 1c).

### 3.2 LINC00853 induced proliferation of gastric cancer cells

**Fig. 2:**
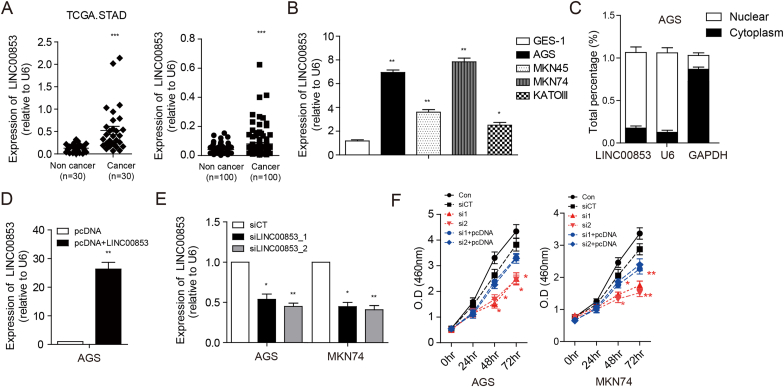
**Expression of LINC00853 was upregulated in gastric cancer cell lines and tissues, and construction of loss and gain function in gastric cancer cells.** (A) LINC00853 expression was first analyzed in STAD-TCGA data (n = 30) and estimated in gastric cancer tissues and adjacent normal tissues (n = 100) by qRT-PCR and calculated by the 2-ΔΔCt method. (B) LINC00853 expression was clearly upregulated in gastric cancer cells (AGS, MKN45, MKN74, and KATOIII) compared to that in normal gastric cells (GES-1). (C) LINC00853 expression in nuclear and cytoplasmic fractions of AGS cells measured by qRT-PCR. LINC00853 expression was measured by qRT-PCR in AGS cells and MKN74 cells transfected with (D) LINC00853 overexpression vector (pcDNA-LINC00853) and (E) two different siRNAs. (F) Cell proliferation was detected by MTS analysis in AGS and MKN74 cell lines. AGS and MKN74 cells transfected by siCT, siLINC00853_1, and siLINC00853_2 (si1 and si2), and siRNAs followed by pcDNA-LINC00853 (si1/2+pcDNA). Error bars represent mean ± SD obtained from three independent experiments. Asterisks indicate statistically significant differences compared to scrambled control group (*P ≤ 0.05, **P ≤ 0.01, ***P ≤ 0.001).

Among the five lncRNAs validated in this study, LINC00853 was significantly upregulated in gastric cancer compared to non-tumor tissues in the genotype-tissue expression (GTEx) and The Cancer Genome Atlas (TCGA) datasets (P ≤ 0.001) (Fig. 2a). We further verified the expression of LINC00853 in 100 patients who had undergone surgical resection for gastric cancer. The expression of LINC00853 was significantly higher in cancer tissues than in non-tumor tissues (P ≤ 0.001) (Fig. 2a). The expression of LINC00853 was significantly elevated in four gastric cancer cell lines (AGS, MKN45, MKN 74, and KATOIII) compared to a normal cell line (GES-1) (Fig. 2b). AGS and MKN74 cells that expressed LINC00853 at relatively high levels were selected for further experiments. LINC00853 was predominantly located in the nucleus rather than in the cytosol of AGS cells (Fig. 2c). To investigate the function of LINC00853 in gastric cancer, overexpression and knockdown systems were developed. The pcDNA LINC00853 overexpression vector resulted in LINC00853 overexpression, which was approximately 28-fold higher than that observed with the pcDNA vector alone (Fig. 2d). Two different siRNAs, siLINC00853_1 and siLINC00853_2, reduced the expression of LINC00853 by approximately 50% compared to that in the AGS and MKN74 cells transfected with siCT (Fig. 2e). Cell proliferation was determined by cell proliferation assay after transfection with the two different siRNAs specific to LINC00853. Both siLINC00853_1 and siLINC00853_2 significantly decreased the viability of AGS (Fig. 2h, red line) and MKN74 (Fig. 2h, red line) cells after 48 h compared to siCT. These reductions were reversed by the restoration of LINC00853 expression by pcDNA-LINC00853 transfection in both the AGS (Fig. 2h, blue line) and MKN74 (Fig. 2h, blue line) cells.

### 3.3 LINC00853 promotes the invasion and migration of gastric cancer cells

**Fig. 3:**
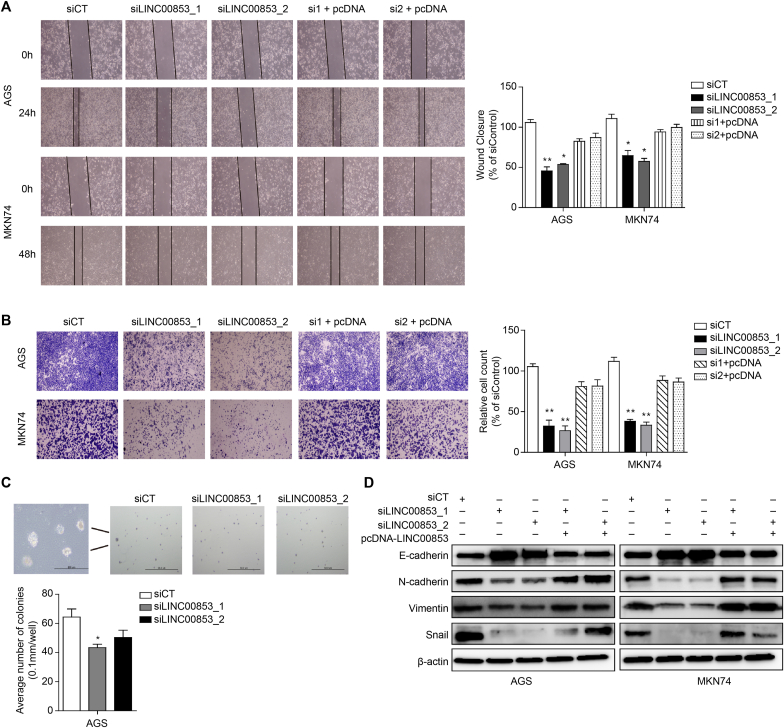
**LINC00853 regulates cell migration, invasion, and colony formation.** (A) Wound-healing, (B) invasion, and (C) colony formation assays were performed by transfecting siCT, siLINC00853, or siLINC00853 followed by pcDNA-LINC00853 in AGS and MKN74 cells. (D) Epithelial–mesenchymal transition markers were determined by Western blotting in the same way. Error bars represent mean ± SD obtained from three independent experiments. si1 and si2 indicate siLINC00853_1 and siLINC00853_2, and pcDNA indicates the LINC00853 overexpression vector. Asterisks indicate statistically significant differences compared to scrambled control group (*P ≤ 0.05, **P ≤ 0.01).

When two different siLINC00853s were transfected, the migration of AGS and MKN74 cells was significantly reduced in the wound-healing analysis (Fig. 3a). In the Matrigel invasion analysis, siLINC00853s inhibited the invasion of AGS and MKN74 cells compared to siCT, which was consistent with results of the migration analysis (Fig. 3b). The decrease in gastric cancer cell invasion and migration by siLINC00853s was largely reversed by transfection with pcDNA-LINC00853 (Fig. 3a and b). To measure the anchorage-independent growth of the cells *in vitro*, we performed a soft agar colony formation analysis. The number and size of colonies were significantly reduced in cells transfected with siLINC00853_1 compared to those transfected with siCT (Fig. 3c). A decrease in the number and size of colonies was observed in cells transfected with siLINC00853_2; however, this difference was not significant (Fig. 3c). We evaluated the differential expression of epithelial–mesenchymal transition markers by Western blot analysis. The expression of the mesenchymal markers Snail, N-cadherin, and Vimentin increased in cells transfected with siLINC00853s compared to cells transfected with siCT, while the expression of E-cadherin decreased. These changes were reversed upon transfection with pcDNA-LINC00853 (Fig. 3d). Therefore, LINC00853 promoted the invasion, migration, and colony formation of gastric cancer cells.

### 3.4 LINC00853 interacts with MAP17 and its downstream pathways

**Fig. 4:**
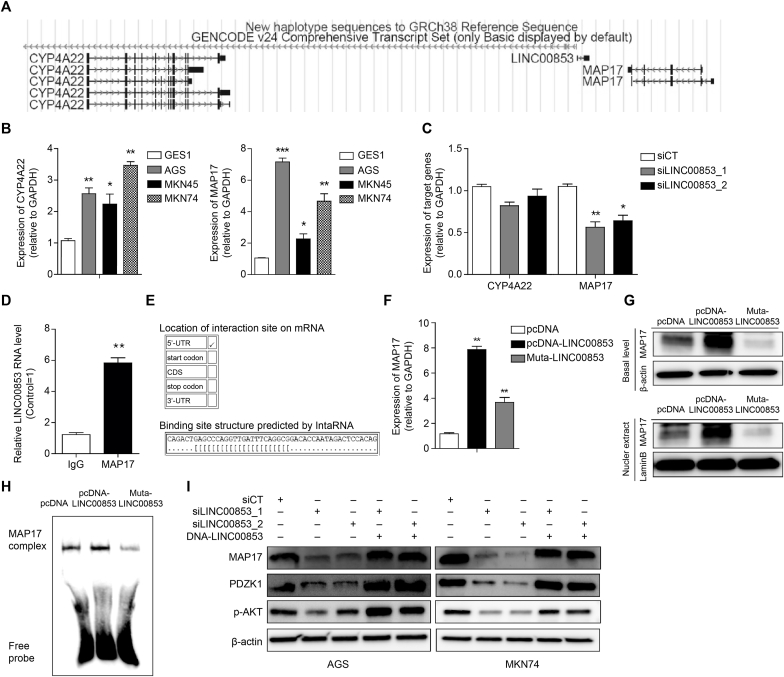
**Cis-regulatory function of LINC00853 regulates the MAP17/PDZK1/AKT pathway.** (A) LINC00853 genomic locus with neighboring genes CYP4A22 and MAP17. (B) Expression levels of CYP4A22 and MAP17 were both upregulated in gastric cancer cell lines compared to normal cells. (C) Expression levels of CYP4A22 and MAP17 were measured by qRT-PCR using LINC00853 knockdown in AGS cells. (D) RIP analysis was performed using anti-MAP17 and anti-IgG in AGS cell lysates. The bar graph shows the relative RNA levels of LINC00853 after anti-MAP17 immunoprecipitation using the lysate. (E) The potential binding sites between LINC00853 and MAP17 were predicted using a web program (//rtools.cbrc.jp/cgi-bin//RNARNA/index.pl). (F) MAP17 RNA expression was measured by qRT-PCR in AGS cells transfected with pcDNA, pcDNA-LINC00853, or LINC00853-Mut induction. (G) AGS cells were exposed to pcDNA, pcDNA-LINC00853, or LINC00853-Mut and then total protein (upper) and nuclear protein (lower) were separated and analyzed by Western blotting. LaminB was used as a loading control. (H) MAP17's DNA binding activity was analyzed by gel shift assay after transfecting with pcDNA, pcDNA-LINC00853, and LINC00853-Mut. (I) Western blot was performed for target gene MAP17 and downstream PDZK/AKT signaling pathway in AGS and MKN74 cells. Error bars represent mean ± SD obtained from three independent experiments. Asterisks indicate statistically significant differences compared to scrambled control group (*P ≤ 0.05, **P ≤ 0.01).

To elucidate the mechanism of regulation of the biological functions of gastric cancer by LINC00853, we analyzed candidate genes that could be regulated by the *cis*-regulatory mechanism of LINC00853. The genomic locations of LINC00853 and its neighboring genes CYP4A22 and MAP17 were displayed using the UCSC Genome Browser (https://genome.ucsc.edu/) (Fig. 4a). The expression of CYP4A22 and MAP17 was moderately upregulated in gastric cancer cell lines compared to that in normal cells (Fig. 4b). Next, we treated AGS cells with siLINC00853s to confirm whether LINC00853 interacted with CYP4A22 and MAP17. Both siLINC00853s significantly reduced the expression of MAP17, whereas the expression of CYP4A22 remained unchanged (Fig. 4c). Microarray data also showed that MAP17 is a downstream target of LINC00853, with positive regulation (Table 3). We performed RIP analysis to determine the interaction between LINC00853 and MAP17, which demonstrated a clear binding interaction between these two factors in the AGS cells (Fig. 4d). We then used a web program (//rtools.cbrc.jp/cgi-bin//RNARNA/index.pl) (Fig. 4e) to predict the binding sites of LINC00853 and MAP17 and established a LINC00853 plasmid vector containing intact binding and mutated binding sites. Cells transfected with the intact LINC00853 plasmid (pcDNA-LINC00853) showed a significant increase in the RNA (Fig. 4f) and protein (Fig. 4g, upper panel) levels of MAP17, whereas cells transfected with mutated LINC00853 (LINC00853-Mut) showed a decrease in MAP17 RNA (Fig. 4f) and protein (Fig. 4g, upper panel) levels. To confirm the binding of MAP17 to LINC00853, which is mainly located in the nucleus, we conducted nuclear/cytoplasmic fractionation and EMSA analysis. A decrease in MAP17 levels was observed in the nuclear extract of the AGS cells transfected with the LINC00853-Mut vector compared to the levels in the control cells and those transfected with pcDNA-LINC00853 (Fig. 4g, bottom). In EMSA, MAP17 probe binding was detected in AGS cells transfected with control or pcDNA-LINC00853, and binding was reduced when transfected with LINC00853-Mut (Fig. 4h). We performed Western blot analysis to observe the effect of LINC00853 on the PDZK1/AKT signaling pathway downstream of MAP17. The protein levels of MAP17, PDZK1, and p-AKT were significantly reduced after siLINC00853 treatment, whereas LINC00853 overexpression had the opposite effect (Fig. 4i).

**Table 3:** Highly expressed lncRNAs in EGC tissues vs. adjacent non-cancer tissues.

gene_id	Genome relationship	Nearby gene symbol	Fold change -mRNA	P-value -mRNA	Regulation- mRNAs
LINC00853	Downstream	PDZK1IP1 (MAP17)	5.3876968	0.0362763	Up
(lncRNA)					

### 3.5 MAP17 and LINC00853 act co-operatively during carcinogenesis associated with gastric cancer

**Fig. 5:**
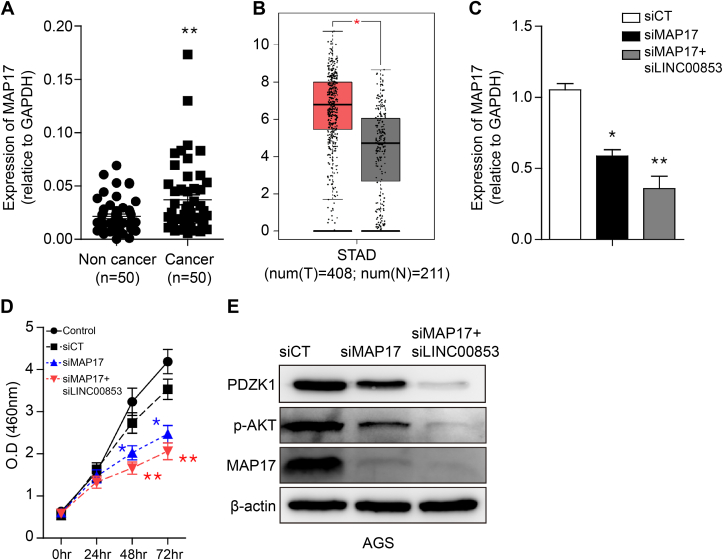
**The effect of MAP17 on gastric tumorigenesis.** (A) Expression of MAP17 estimated in gastric tissues and adjacent normal tissues (n = 50) using qRT-PCR and calculated by 2-ΔΔCt method. (B) Expression of MAP17 measured in STAD-TCGA data. (C) Loss of function with MAP17 expression was evaluated by qRT-PCR. Expression of MAP17 was analyzed using AGS cells transfected with siCT, siMAP17, and double knockdown of MAP17 and LINC00853. (D) Cell viability was assessed using an MTS assay. (E) Western blot analysis was performed in the products of the LINC00853 target gene (MAP17) and its downstream proteins (PDZK1 and AKT). Error bars represent the mean ± SD from three independent experiments. Asterisks indicate statistically significant differences compared to scrambled control group (*P ≤ 0.05, **P ≤ 0.01).

MAP17 has been shown to act as an oncogene in various cancers [[39], [40], [41]]. To elucidate the role of MAP17 in gastric cancer, we measured its mRNA expression in 50 patients with gastric cancer. MAP17 was significantly upregulated in cancer tissues compared to adjacent non-cancerous tissues (Fig. 5a). In the box plot of the STAD-TCGA data, MAP17 expression was upregulated in gastric cancer tissues compared to normal tissues (http://gepia.cancer-pku.cn/detail.php?gene=MAP17#iframe) (Fig. 5b). Next, we developed an siRNA against MAP17 (siMAP17), which showed that MAP17 expression was reduced by approximately 50% in AGS cells. Double knockdown of MAP17 and LINC00853 resulted in a more pronounced decrease in MAP17 expression than single knockdown of MAP17 (Fig. 5c). In addition, in the MTT assay, double knockdown of MAP17 and LINC00853 showed greater inhibition of AGS cell proliferation than single knockdown of MAP17 (Fig. 5d). To observe the effect of the double knockdown of MAP17 and LINC00853 on the PDZK1/AKT signaling pathway, we performed Western blotting. Downstream signaling pathways, including p-AKT and PDZK1, were downregulated by the single knockdown of MAP17, and double knockdown mediated a further decrease in the expression of these targets (Fig. 5e). Therefore, MAP17 and LINC00853 co-operatively promote carcinogenic activity and signaling pathways associated with gastric cancer.

### 3.6 Exosome-mediated transfer of LINC00853 induces gastric cancer progression via MAP17 upregulation

**Fig. 6:**
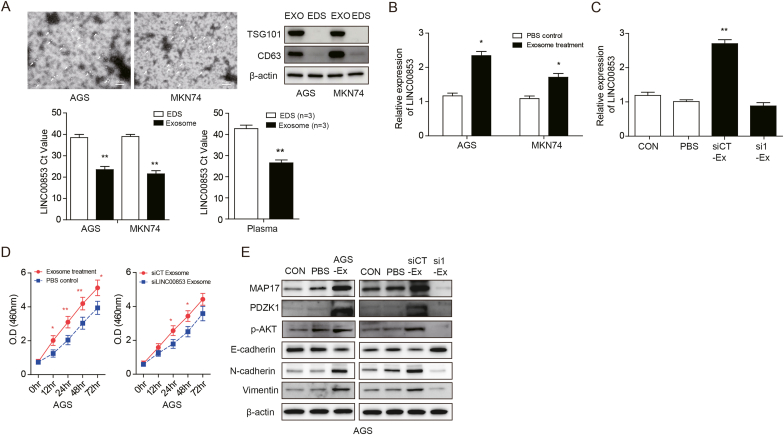
**Exosome-mediated transfer of LINC00853 induces gastric cancer progression via MAP17 upregulation.** (A) (Upper panel) Representative TEM image of Exosomes. Scale bar: 100 nm. Western blotting analysis of exosomal markers, TSG101 and CD63. (Lower panel) LINC00853 expression levels in exosomes derived from depleted serum and patient plasma samples (n = 3) and gastric cancer cell lines. (B) qRT-PCR analysis of exosomal LINC00853 expression levels in two different gastric cancer cells treated with extracted exosomes or PBS control. (C) qRT-PCR analysis of exosomal LINC00853 expression in AGS cells treated with PBS (PBS), exosomes extracted from AGS cells or exosomes from AGS cells transfected with siCT (siCT-Ex), and siLINC00853 (si1-Ex). (D) Cell proliferation analysis in AGS cells treated as in (C) from 0 to 72 h. (E) Western blot analysis of epithelial–mesenchymal transition markers, and MAP17, PDZK1, and p-AKT expression in AGS cells treated as in (C). Con: Control, AGS-Ex: Exosomes extracted from AGS cells. Error bars represent mean ± SD obtained from three independent experiments. Asterisks indicate statistically significant differences compared to PBS control group or scrambled control group (*P ≤ 0.05).

To confirm the aberrant expression of exosomal LINC00853 in gastric cancer, we collected plasma samples from three patients and culture medium from AGS gastric cancer cells and examined the EXO and EXO-depleted supernatant (EDS). To verify the separation of exosomes, we utilized Transmission Electron Microscopy (TEM) to observe the exosomes. To confirm the complete separation of EXO and EDS, we performed a Western blot analysis, which demonstrated a dominant expression of exosomal markers TSG101 and CD63 in EXO compared to EDS (Fig. 6a, upper panel). LINC00853 was found to be abundantly expressed in EXO as investigated by qRT-PCR (Fig. 6a, lower panel). Next, we tested whether EXO affected the expression of LINC00853 in AGS and MKN74 cells. The cell lines were treated with EXO extracted from gastric cancer cell medium and PBS for 24 h. Treatment with the extracted EXO resulted in a significant increase in LINC00853 expression in the AGS and MKN74 cells compared to that in the PBS control (Fig. 6b). LINC00853 expression induced by EXO treatment was more pronounced in AGS cells than in MKN74 cells. After transfection with siCT or siLINC00853, we extracted the EXO from the AGS cells. Subsequently, the AGS cells were treated with these EXO for 24 h, and LINC00853 levels were measured using qRT-PCR (Fig. 6c). Treatment with EXO derived from siLINC00853-treated cells resulted in a significant decrease in LINC00853 expression in AGS cells compared to that observed in cells cultured with siCT (Fig. 6c). We also examined whether LINC00853 in the EXO affected the proliferation of gastric cancer cells. As expected, when treated with EXO derived from the AGS culture medium, AGS cell proliferation was significantly induced after 12 h of culture compared with that observed in the PBS control (Fig. 6d, left). However, EXO from siLINC00853-treated AGS cells significantly reduced AGS cell proliferation after 24 h of culture compared to EXO derived from siRNA-treated AGS cells (Fig. 6d, right). Therefore, siRNA-mediated knockdown of LINC00853 resulted in decreased LINC00853 expression in EXO, which aggravated the oncogenic effect of exosomal LINC00853 in gastric cancer cells. Western blot analysis showed that EXO treatment induced the expression of N-cadherin, Vimentin, MAP17, PDZK1, and p-AKT and downregulated the expression of E-cadherin. However, EXO derived after LINC00853 knockdown significantly reversed this effect (Fig. 6e).

## 4 Discussion

In this study, we performed lncRNA microarray analysis on EGC tissues and adjacent non-cancerous tissues from patients with single and synchronous EGC to identify the lncRNAs involved in the onset and recurrence of EGC. After selecting eight differentially expressed lncRNAs, five lncRNAs—LINC00853, LINC00634, LINC01535, GAPLINC, and AC017002.1—were validated in samples from patients with EGC (Fig. 1). Among them, LINC00853 was the most important lncRNA that showed significantly increased expression in EGC and advanced gastric cancer samples compared to non-tumor tissues in endoscopic biopsy samples collected from our institute and the GTEx and TCGA datasets (Table 3). LINC00853 facilitated the proliferation, epithelial–mesenchymal transition, invasion, and migration of gastric cancer cells (Fig. 2, Fig. 3). These oncogenic effects of LINC00853 were confirmed to be owing to the activation of the PDZK1/AKT signaling pathway through binding to the MAP17 promoter located near LINC00853 (Fig. 4). To identify potential molecular targets and biomarkers for EGC progression, we searched for lncRNAs extracted from patient plasma samples and EXO extracted from gastric cancer cell culture media. We investigated the functional correlation between gastric tumor formation and exosomal LINC00853. LINC00853 was secreted as a component of EXO in gastric cancer, and exosomal LINC00853 exerted oncogenic effects through MAP17-related cell signaling pathways in gastric cancer cells (Fig. 6). To our knowledge, this is the first study to report the functional roles of exosomal and cellular LINC00853 in EGC development. We confirmed the active function of exosomal LINC00853 in gastric carcinogenesis, suggesting that exosomal LINC00853 might be a candidate for targeted therapy, as well as a diagnostic biomarker.

EXO, which are a subset of small extracellular vesicles produced by various cells, have attracted great attention as biomarkers for various diseases, including cancers, owing to their role in intercellular communication [27,30]. EXO comprise lipoproteins that can deliver cellular components, including mRNA, miRNAs, and lncRNAs [42,43]. LncRNAs delivered via EXO are involved in mediating host cell migration, invasion, proliferation [42,44,45], chemoresistance [31,46], stemness [47], and immunotherapy in various cancers. Recently, researchers have reported that serum extracellular vesicle–derived LINC00853 (EV-LINC00853) can be used as a diagnostic biomarker for early hepatocellular carcinoma [48]. In their study, they found seven lncRNAs that were differentially expressed between hepatocellular carcinoma and non-tumor tissues using TCGA data. They found that only the expression of EV-LINC00853 increased in hepatocellular carcinoma, and that EV-LINC00853 showed excellent discrimination in pre-stage disease diagnosis. The accuracy of EV-LINC00853 was more evident in hepatocellular carcinoma, where the traditional serum biomarker alpha-fetoprotein was not expressed. Unfortunately, the oncogenic mechanism of LINC00853 in hepatocellular carcinoma was not investigated in their study. However, we were able to confirm that LINC00853 has a clear oncogenic mechanism in gastric cancer. Combining the results of our study with those of previous studies suggests that exosomal LINC00853 could be used as a biomarker for gastric cancer. Here, all the oncogenic effects of LINC00853, with its neighboring gene MAP17, seemed to be exerted through the MAP17/PDZK1/AKT signaling pathway. Thus, LINC00853 and MAP17 may act as co-operative oncogenes in gastric cancer.

LncRNAs play a regulatory role through *cis*-action, influencing the transcription of neighboring genes. Additionally, they can undergo *trans*-action, engaging in various cellular activities beyond neighboring genes or chromatin, which, in turn, affects the structure of the cell nucleus, as well as proteins and RNA [49]. Through *cis*-regulatory mechanisms, functional genetic regulatory elements, such as enhancers located within lncRNAs, often modulate the expression of adjacent genes [50]. MAP17 is a neighboring gene of LINC00853 and is a representative oncogene mainly expressed in various cancers [39,40]. We found that LINC00853 regulated the expression of MAP17 and bound to its promoter region. PDZK1/AKT/E-CAD is a downstream signaling pathway of MAP17 [51] and is one of the signaling pathways involved in gastric cancer. Among the candidate lncRNAs, the functions of LINC00853 have been observed in other types of cancer. Moreover, LINC00634 exerts oncogenic effects in esophageal squamous cell carcinoma via the miR-342-3p/Bcl2L1 axis [52], while LINC01535 promotes cervical cancer by targeting the miR-214–EZH2 feedback loop [53]. In addition, GAPLINC is a gastric adenocarcinoma–related lncRNA that positively regulates CD44 in various cancers [54]. Our findings, in combination with previous observations, emphasize the broader implications of understanding these lncRNAs across various cancer contexts, prompting the need for further comprehensive investigations.

This study has some limitations. Exosomal LINC00853 showed potential as a biomarker for EGC; however, we were unable to confirm these results using actual EGC samples. Therefore, patient samples and tissues should be analyzed in future studies. In conclusion, our study confirmed that exosomal LINC00853 is a potential biomarker of gastric cancer and revealed its regulatory effects on the MAP17/PDZK1/AKT pathway in gastric cancer.

## CRediT authorship contribution statement

**Jung-ho Yoon:** Writing – review & editing, Resources, Methodology, Investigation. **Hyo Joo Byun:** Writing – original draft, Validation, Software, Methodology, Formal analysis, Data curation. **Seo Yeon Kim:** Validation. **Da Hyun Jung:** Visualization, Software. **Sang Kil Lee:** Writing – review & editing, Supervision, Resources, Project administration, Funding acquisition.

## Declaration of competing interest

The authors declare that they have no known competing financial interests or personal relationships that could have appeared to influence the work reported in this paper.

## References

[1] Bertuccio P. (2009). Recent patterns in gastric cancer: a global overview. Int. J. Cancer.

[2] Ferro A. (2014). Worldwide trends in gastric cancer mortality (1980-2011), with predictions to 2015, and incidence by subtype. Eur. J. Cancer.

[3] Iascone C. (2013). Early gastric cancer: an overview and future perspective. J. Gastrointest. Dig. Syst. 01.

[4] McLean M.H., El-Omar E.M. (2014). Genetics of gastric cancer. Nat. Rev. Gastroenterol. Hepatol..

[5] Choi S.R. (2006). Role of serum tumor markers in monitoring for recurrence of gastric cancer following radical gastrectomy. Dig. Dis. Sci..

[6] Wu H.H., Lin W.C., Tsai K.W. (2014). Advances in molecular biomarkers for gastric cancer: miRNAs as emerging novel cancer markers. Expet Rev. Mol. Med..

[7] Esteller M. (2011). Non-coding RNAs in human disease. Nat. Rev. Genet..

[8] Peng W.X., Koirala P., Mo Y.Y. (2017). LncRNA-mediated regulation of cell signaling in cancer. Oncogene.

[9] Yang G., Lu X., Yuan L. (2014). LncRNA: a link between RNA and cancer. Biochim. Biophys. Acta.

[10] Ponting C.P., Oliver P.L., Reik W. (2009). Evolution and functions of long noncoding RNAs. Cell.

[11] Byun H.J., Yoon J.H., Lee S.K. (2020). LUCAT1 epigenetically downregulates the tumor suppressor genes CXXC4 and SFRP2 in gastric cancer. Yonsei Med. J..

[12] Seo S.I., Yoon J.H., Byun H.J., Lee S.K. (2021). HOTAIR induces methylation of PCDH10, a tumor suppressor gene, by regulating DNMT1 and sponging with miR-148b in gastric adenocarcinoma. Yonsei Med. J..

[13] Ali M.M. (2018). Pan-cancer analysis of S-phase enriched lncRNAs identifies oncogenic drivers and biomarkers. Nat. Commun..

[14] Ge X. (2013). Overexpression of long noncoding RNA PCAT-1 is a novel biomarker of poor prognosis in patients with colorectal cancer. Med. Oncol..

[15] Gupta R.A. (2010). Long non-coding RNA HOTAIR reprograms chromatin state to promote cancer metastasis. Nature.

[16] Carl-McGrath S., Eabert M., Rocken C. (2007). Gastric adenocarcinoma: epidemiology, pathology and pathogenesis. Cancer Ther..

[17] Tan P., Yeoh K.G. (2015). Genetics and molecular pathogenesis of gastric adenocarcinoma. Gastroenterology.

[18] Zou X.P. (2009). Promoter hypermethylation of multiple genes in early gastric adenocarcinoma and precancerous lesions. Hum. Pathol..

[19] Lee J.H. (2004). Frequent CpG island methylation in precursor lesions and early gastric adenocarcinomas. Oncogene.

[20] Yu J. (2009). Methylation of protocadherin 10, a novel tumor suppressor, is associated with poor prognosis in patients with gastric cancer. Gastroenterology.

[21] Shi J. (2012). Prognostic significance of aberrant gene methylation in gastric cancer. Am. J. Cancer Res..

[22] Maeda M. (2017). High impact of methylation accumulation on metachronous gastric cancer: 5-year follow-up of a multicentre prospective cohort study. Gut.

[23] Okugawa Y. (2014). Metastasis-associated long non-coding RNA drives gastric cancer development and promotes peritoneal metastasis. Carcinogenesis.

[24] Zhao J. (2015). Long non-coding RNA Linc00152 is involved in cell cycle arrest, apoptosis, epithelial to mesenchymal transition, cell migration and invasion in gastric cancer. Cell Cycle.

[25] Ma M. (2018). lncRNA GCAWKR promotes gastric cancer development by scaffolding the chromatin modification factors WDR5 and KAT2A. Mol. Ther..

[26] Zhao J. (2018). LncRNA PVT1 promotes angiogenesis via activating the STAT3/VEGFA axis in gastric cancer. Oncogene.

[27] Dai J. (2020). Exosomes: key players in cancer and potential therapeutic strategy. Signal Transduct. Targeted Ther..

[28] Nam G.H. (2020). Emerging prospects of exosomes for cancer treatment: from conventional therapy to immunotherapy. Adv. Mater..

[29] Dragomir M., Chen B., Calin G.A. (2018). Exosomal lncRNAs as new players in cell-to-cell communication. Transl. Cancer Res..

[30] Sun Z. (2018). Emerging role of exosome-derived long non-coding RNAs in tumor microenvironment. Mol. Cancer.

[31] Zang X. (2020). Exosome-transmitted lncRNA UFC1 promotes non-small-cell lung cancer progression by EZH2-mediated epigenetic silencing of PTEN expression. Cell Death Dis..

[32] Wang J. (2019). Exosome-mediated transfer of lncRNA HOTTIP promotes cisplatin resistance in gastric cancer cells by regulating HMGA1/miR-218 axis. OncoTargets Ther..

[33] Du Z. (2013). Integrative genomic analyses reveal clinically relevant long noncoding RNAs in human cancer. Nat. Struct. Mol. Biol..

[34] Xu N., Wang F., Lv M., Cheng L. (2015). Microarray expression profile analysis of long non-coding RNAs in human breast cancer: a study of Chinese women. Biomed. Pharmacother..

[35] Jia J. (2019). LncRNA H19 interacted with miR-130a03p and miR-17-5p to modify radio-resistance and chemo-sesitivity of cardiac carcinoma cells. Cancer Med..

[36] Zhu Y.P. (2014). Long noncoding RNA expression signatures of bladder cancer revealed by microarray. Oncol. Lett..

[37] Liu W.T. (2014). LncRNAs expression signatures of hepatocellular carcinoma revealed by microarray. World J. Gastroenterol..

[38] Shang Z. (2019). LncRNA PCAT1 activates AKT and NF-κB signaling in castration-resistant prostate cancer by regulating the PHLPP/FKBP51/IKKα complex. Nucleic Acids Res..

[39] Carnero A. (2012). MAP17, a ROS-dependent oncogene. Front. Oncol..

[40] Guijarro M.V. (2007). MAP17 inhibits Myc-induced apoptosis through PI3K/AKT pathway activation. Carcinogenesis.

[41] Muñoz-Galván S., Gutierrez G., Perez M., Carnero A. (2015). MAP17 (PDZKIP1) expression determines sensitivity to the proteasomal inhibitor bortezomib by preventing cytoprotective autophagy and NF-kappaB activation in breast cancer. Mol. Cancer Therapeut..

[42] Chen L. (2017). Exosomal lncRNA GAS5 regulates the apoptosis of macrophages and vascular endothelial cells in atherosclerosis. PLoS One.

[43] Luo X. (2019). Exosomal lncRNA HNF1A-AS1 affects cisplatin resistance in cervical cancer cells through regulating microRNA-34b/TUFT1 axis. Cancer Cell Int..

[44] Chen C. (2020). Exosomal long noncoding RNA LNMAT2 promotes lymphatic metastasis in bladder cancer. J. Clin. Invest..

[45] Gao T. (2018). Exosomal lncRNA 91H is associated with poor development in colorectal cancer by modifying HNRNPK expression. Cancer Cell Int..

[46] Kang M. (2018). Exosome-mediated transfer of lncRNA PART1 induces gefitinib resistance in esophageal squamous cell carcinoma via functioning as a competing endogenous RNA. J. Exp. Clin. Cancer Res..

[47] Ramteke A. (2015). Exosomes secreted under hypoxia enhance invasiveness and stemness of prostate cancer cells by targeting adherens junction molecules. Mol. Carcinog..

[48] Kim S.S. (2020). Serum small extracellular vesicle-derived LINC00853 as a novel diagnostic marker for early hepatocellular carcinoma. Mol. Oncol..

[49] Kopp F., Mendell J.T. (2018). Functional classification and experimental dissection of long noncoding RNAs. Cell.

[50] Gil N., Ulitsky I. (2020). Regulation of gene expression by cis-acting long non-coding RNAs. Nat. Rev. Genet..

[51] Ikeno S. (2019). PDZK1-interacting protein 1 (PDZK1IP1) traps Smad4 protein and suppresses transforming growth factor-beta (TGF-beta) signaling. J. Biol. Chem..

[52] Zhang X., Feng Y., Gao Y., Hu J. (2020). Long noncoding RNA LINC00634 functions as an oncogene in esophageal squamous cell carcinoma through the miR-342-3p/Bcl2L1 axis. Technol. Cancer Res. Treat..

[53] Song H. (2019). Long non-coding RNA LINC01535 promotes cervical cancer progression via targeting the miR-214/EZH2 feedback loop. J. Cell Mol. Med..

[54] Hu Y. (2014). Long noncoding RNA GAPLINC regulates CD44-dependent cell invasiveness and associates with poor prognosis of gastric cancer. Cancer Res..

